# Novel Presentation of Sturge–Weber Syndrome in a Boy With a Port-Wine Birthmark

**DOI:** 10.1155/crpe/6665247

**Published:** 2025-04-25

**Authors:** Barbara Anna Folga, Ramzan Shahid

**Affiliations:** Department of Pediatrics, Loyola University Medical Center, Maywood 60153, Illinois, USA

## Abstract

Sturge–Weber Syndrome (SWS), also called encephalotrigeminal angiomatosis, is a rare congenital neurological condition classified by the hallmark findings of a port-wine birthmark, leptomeningeal angiomas, and glaucoma. Here, we present a case of a two-year-old patient with a history of a left-sided port-wine birthmark involving the V1-V2 dermatomes who re-presented to the emergency department with focal right-sided seizure-like activity in the setting of a recent head trauma. The patient was admitted for further workup, where video electroencephalography demonstrated the presence of electrographic seizures developing from the left posterior quadrant, with continuous focal slowing over the left hemisphere, and with magnetic resonance venography revealing findings concerning for a pial angiomatosis. This finding, coupled to new-onset seizure-like activity in a patient with a port-wine birthmark, supported a diagnosis of SWS. He was started on an antiepileptic drug regimen with resolution of seizure-like activity while inpatient and his subsequent care was transferred to a specialized clinic designed to manage patients with SWS. Most patients diagnosed with SWS exhibit seizure-like activity, often presenting as infantile spasms, within the first year of life; our patient, on the other hand, began to exhibit focal seizures following a traumatic event and was later found to harbor findings consistent with SWS. Overall, this case highlights the role of a multidisciplinary team in the management of patients with SWS and demonstrates the importance of routine follow-up testing, imaging, and subspecialty care for these patients.

## 1. Introduction

Sturge–Weber Syndrome (SWS) is a rare congenital neurological condition characterized by a triad of a port-wine birthmark, glaucoma, and leptomeningeal angiomas. It is diagnosed based on symptomatology, physical examination, and imaging findings [1]. In addition to the leptomeningeal enhancements and pial angiomatosis demonstrated on magnetic resonance imaging (MRI), patients with SWS may exhibit subcortical calcifications on computer tomography (CT), often observed in a “tram-track”-like appearance [2]; these are common and evolve rapidly in pediatric patients with SWS who develop epilepsy early on [3]. The number and severity of these calcifications is linked to more severe and significant epilepsy and seizure burden [4].

Infantile spasms are the most common manifestation of seizure-like activity in patients with SWS within the first year of life. After experiencing infantile spasms, these patients oftentimes develop atonic, tonic or myoclonic seizures [1], which have been linked to progressively worsening hemiparesis and increasing resistance to treatment modalities [5]. Less commonly, children may present with a gaze preference or with early handedness [6]. Moreover, fevers and infection have been implicated as triggers of new-onset seizure-like activity in patients with SWS [7].

Patients with SWS may also experience stroke-like episodes, marked by transitory periods of hemiparesis, with or without difficulties in speech [8], related to abnormal venous drainage and arterial perfusion in affected brain regions, as revealed on perfusion imaging scans [9]. These often have a variable clinical course with variable duration. Due to the nature of these episodes, patients with SWS may be placed on low-dose aspirin, which has been shown to reduce the number of stroke-like episodes and seizure symptoms in this population [10].

## 2. Case Presentation

Here, we present a case of a two-year-old male patient born full-term with a history of a left-sided port wine birthmark involving the V1-V2 dermatomes who re-presented to the emergency department (ED) with new-onset focal right-sided seizure-like activity in the setting of a head injury which had transpired 6 days prior. Preceding the head injury, the patient was noted to be in his usual state of health; the morning of the accident, the patient was roughhousing with his elder sibling when they collided heads. The patient did not lose consciousness. About thirty minutes after the collision, the patient was noted to be more tired than usual and experienced a few episodes of emesis. Later on that day, he was evaluated at the ED, where he was noted to be in no acute distress, alert, without focal neurological deficits observed. CT head returned negative for an acute intracranial abnormality. While there, he failed a per os (PO) challenge, was offered hospital admission for observation but parents declined, after which point he was then discharged with strict return precautions. The following day, the patient was evaluated at his pediatrician's office, where he was alert, active, with a steady and balanced gait, 5/5 muscle strength and with cranial nerves (CN) II-XII intact. In the days preceding this office visit, the patient's symptoms were improving, though he complained of a headache and experienced several witnessed, brief (lasting between 5 and 10 s), self-resolved staring spells to the right. These were not associated with a prodrome, convulsions, or a postictal period, and the patient returned to his baseline following these episodes. Five days after the collision, the patient experienced one prolonged staring episode, at which point it took the patient a longer period of time to react, but these symptoms were self-limited. Six days after the injury, the patient's father awoke to find his son's right upper and lower extremity shaking, describing it as a jerking motion with associated drooling, abnormal eye rolling movements, and cheek contractions. Emergency medical services were notified, who, upon arrival, administered one dose of intranasal midazolam with symptom resolution; the episode lasted about twenty minutes and was associated with about a ninety minute postictal period. The patient was brought to the ED for further evaluation.

Upon arrival to the ED, the patient's initial set of vital signs were unremarkable. He was noted to be in no acute distress but favored the left side of his body, exhibiting difficulties in moving his right upper and lower extremities and turning his head and neck to the right side. Baseline laboratories were largely within normal limits. A CT scan of the head without intravenous (IV) contrast was obtained and returned negative for an acute intracranial abnormality. The pediatric neurology service was consulted, who recommended admission for further workup, which included placement of a video electroencephalogram (vEEG) and MRI, as this imaging study was not obtained prior to hospitalization.

**Figure 1:**
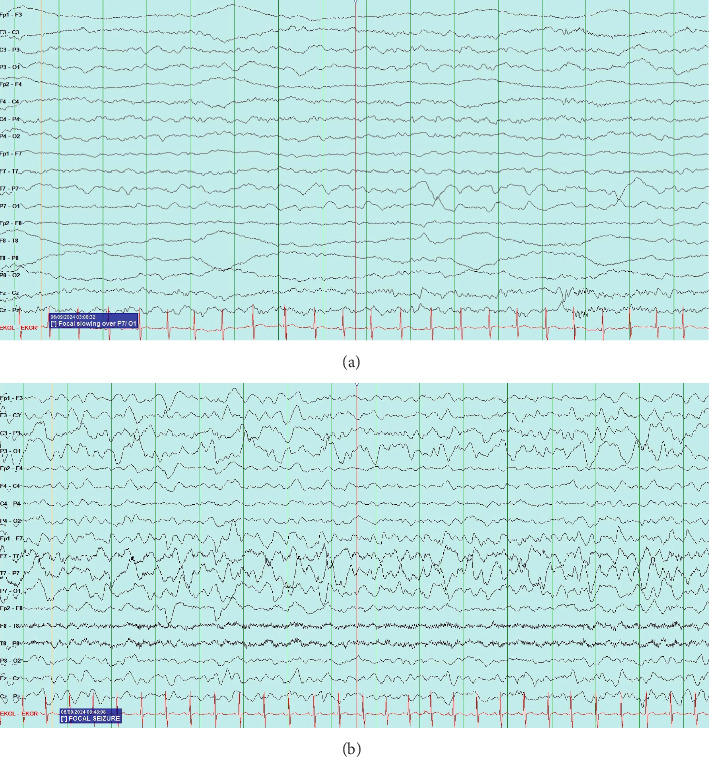
(a) Focal slowing on vEEG. Continuous focal slowing is present over the left hemisphere, maximal in the left posterior quadrant. This finding is suggestive of a structural or functional lesion. (b) Focal seizure on vEEG. Sharp waves demonstrated in the left posterior temporal region. Electrographic seizures are observed developing from the left posterior quadrant.

**Figure 2:**
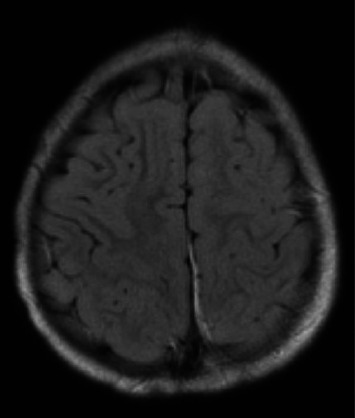
MRI head without IV contrast. Abnormal signal in the left occipital lobe may represent a venous anomaly and chronic blood products but other etiologies not excluded.

**Figure 3:**
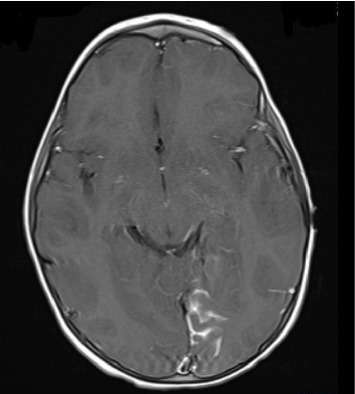
MRI head venogram with and without IV contrast. Sulcal FLAIR hyperintensity in the left paramidline occipital lobe demonstrating leptomeningeal enhancement, most concerning for a pial angiomatosis in a patient with port wine stain. Constellation of findings is worrisome for Sturge–Weber syndrome.

At the time of initial evaluation by the pediatric team in the ED, the patient was able to move the right side of his body but was noted to have mildly decreased tone and strength on this side. Following this assessment, he was admitted to the pediatrics unit and was placed under seizure precautions. vEEG leads were placed soon after arrival. After about 6 hours of recording, continuous focal slowing over the left hemisphere was noted, as demonstrated in Figure 1(a). Between five and nine hours of recording, four electrographic seizures were observed, arising from the left posterior quadrant. Due to these events, the patient was initially started on oxcarbazepine, but because of four further seizure-like episodes, characterized clinically by lip smacking and illustrated in Figure 1(b), on day two of the recording (between thirty and thirty three hours of recording) during which time lorazepam was administered with seizure resolution, the antiepileptic drug (AED) regimen was expanded to include levetiracetam. Seizure activity aborted with this combination and he remained seizure-free for the remainder of his hospitalization. Due to the specific seizure focus, further imaging studies were obtained, and MRI and magnetic resonance venography (MRV) results are outlined in Figures 2 and 3, respectively. Based on these imaging results, in combination with the patient's port-wine birthmark, a diagnosis of SWS was established. The patient was also noted to exhibit an uncoordinated gait that arose during his hospitalization, likely due to hemiparesis in the setting of postictal Todd's paralysis, which improved during his hospitalization. He was discharged on an oxcarbazepine and levetiracetam AED regimen with strict follow-up appointments with pediatric neurology and physical therapy. His subsequent care was transferred to a pediatric center focused on treating patients with SWS.

## 3. Discussion

Unlike in many patients with SWS, who develop seizure-like activity within the first year of life, our patient's seizures were preceded by a traumatic event and occurred after the mean age of onset, symbolizing a novel presentation of SWS in a patient with a port-wine birthmark. Furthermore, the patient initially exhibited what appeared to be staring spells, which later evolved into focal seizure-like activity, representing an unconventional manner in which seizure-like activity in patients with SWS transpires, as most individuals present with infantile spasms. On vEEG, infantile spasms are evidenced by the characteristic finding of hypsarrhythmia, defined as a disorganized brain wave pattern with asynchronous high-amplitude slowing and associated multifocal spike and sharp wave discharges, varied in time [11]. In contrast, the focal seizure-like activity on our patient's vEEG study was rhythmic and organized. Subcortical calcifications were also not evidenced on CT imaging in our patient's case, contributing to the novelty with which this condition emerged.

Despite the fact that many individuals with SWS experience stroke-like episodes, based on the neurology team's assessment, our patient did not exhibit these types of episodes during his hospitalization. Yeom and colleagues suggest initiating low-dose aspirin in patients under the age of three if three or more lobes of the brain are involved [10]; in our patient case, the left occipital lobe was implicated as the source for the patient's clinical symptoms, and he was not started on low-dose aspirin at the time of his hospitalization, but initiation of this medication can be considered in the future depending on the patient's clinical and seizure status. Furthermore, while inpatient, vEEG findings were consistent with electrographic seizures; these resolved with a multi-AED regimen and the patient remained seizure-free posthospital discharge.

In addition to facing stroke-like episodes, patients with SWS also face headaches. These are oftentimes migraines but can rarely persist as tension-type headaches [12]. Headaches that are prolonged and that are accompanied by visual or sensory-motor aura can be specified as a type of stroke-like episode [10]. Our patient's headaches were likely multifactorial in etiology, stemming from his injury, vascular malformation, and the onset of seizures, but they resolved prior to hospital admission. The presence or absence of auras was difficult to assess, owing to the patient's age and developmental status.

## 4. Conclusion

Overall, this case illustrates a rare and atypical presentation of SWS in a patient with a port-wine birthmark and demonstrates the importance of routine follow up testing, imaging, and subspecialty care for patients with SWS, especially for those who present in a unique way. In the absence of evidence of increased intraocular pressure, these patients should follow with an ophthalmologist annually to monitor for the development of glaucoma [1], in addition to regularly scheduled visits with their primary care physician to screen for developmental delays, as the condition has been linked to behavioral difficulties and intellectual deficits [7]. This case also highlights the role of a multidisciplinary team in the management of complex patients with underlying congenital disorders. While there is no cure for SWS, this interprofessional team outlines a treatment plan, one which is designed to control seizure-like activity and slow the progression of complications such as glaucoma. For those patients with treatment refractory epilepsy, surgery, specifically a hemispherectomy or a resection of the epileptogenic zone, may be considered [1]. However, in recent years, there have been efforts to presymptomatically treat patients with SWS prior to the emergence of seizures; while this approach appears promising, more studies need to be completed [13], ideally accounting for patients such as ours in their study design.

## Data Availability

Data sharing is not applicable to this article as no new data were created or analyzed in this study.
